# Advances in Metabolic Reprogramming and Immune Regulatory Mechanisms in Lung Cancer

**DOI:** 10.32604/or.2026.076176

**Published:** 2026-03-23

**Authors:** Xiaomeng Li, Xuejiao Li, Hongbo Wu, Rui Li

**Affiliations:** 1 Department of Respiratory Intervention, The Affiliated Cancer Hospital of Zhengzhou University & Henan Cancer Hospital, No. 127, Dongming Road, Jinshui District, Zhengzhou, China; 2 Department of Basic Medicine Science, North Henan Medical University, Xinxiang, China

**Keywords:** Lung cancer, metabolic reprogramming, immune evasion, tumor microenvironment, metabolic-immune axis

## Abstract

Lung cancer remains the leading cause of cancer-related mortality worldwide, primarily driven by metabolic reprogramming and immune evasion mechanisms within tumor cells. To adapt to the nutrient-deprived tumor microenvironment (TME), lung cancer cells undergo profound metabolic reprogramming, characterized by enhanced glycolysis (the Warburg effect), increased glutamine dependency (mediated by GLS1), and accelerated lipid synthesis (involving enzymes such as FASN). These metabolic alterations not only remodel the TME but also dampen antitumor immune responses by promoting immunosuppressive cell populations (e.g., Tregs and M2 macrophages) and inhibiting effector functions of CD8^+^ T cells and natural killer (NK) cells. Critically, a bidirectional crosstalk operates between tumor cell metabolism and the immunosuppressive TME: metabolic reprogramming drives immune suppression through metabolite accumulation, whereas the immunosuppressive TME, in turn, promotes tumor cell adaptability—thus forming a positive feedback loop that reinforces immune evasion and therapy resistance. This review elucidates key molecular pathways governing metabolic reprogramming in lung cancer—spanning glucose, amino acid, and lipid metabolism—and their dynamic crosstalk with immune regulation, including epigenetic modifications and non-coding RNA-mediated mechanisms. Additionally, it evaluates emerging therapeutic strategies targeting the metabolic-immune axis, such as inhibitors of HK2 or GLS1 combined with anti-PD-1/PD-L1 agents, which aim to reverse immunosuppression and improve clinical outcomes. By synthesizing recent advances, this work provides a theoretical framework for precision oncology interventions, highlighting the potential of metabolic immunotherapies and future directions integrating AI and multi-omics data to overcome resistance in lung cancer.

## 1 Introduction

Lung cancer (LC) remains one of the most frequently diagnosed malignancies worldwide, accounting for approximately 1.76 million deaths annually [1]. In developed countries, long-term tobacco control policies have stabilized or reduced male lung cancer incidence, while female incidence continues to rise due to factors like secondhand smoke and air pollution. In developing countries, persistently high smoking rates, industrial pollution, occupational exposure (asbestos, arsenic compounds), and indoor coal burning pollution have driven continued increases in incidence. Non-small cell lung cancer (NSCLC) accounts for approximately 85% of all lung cancer cases, while small cell lung cancer (SCLC) accounts for 15%. Overall, lung cancer patients have a poor prognosis, which is regulated by multiple factors—including stage, pathological type, molecular characteristics, and patient status—and shows significant heterogeneity.

Lung cancer treatment has evolved from the traditional single-modality approach of surgery plus chemoradiotherapy to individualized multimodal therapy, with core goals of extending survival and improving quality of life. For early-stage disease, treatment centers on radical surgery. Unresectable stage III NSCLC relies on concurrent chemoradiotherapy (CCRT); the PACIFIC regimen (CCRT followed by durvalumab, a PD-L1 inhibitor) boosts 5-year survival from 29% to 42.9%, establishing it as the new standard. Limited-stage SCLC uses CCRT as the only potentially curative option (>95% technical success, 20%–25% 5-year survival). Extensive-stage disease employs chemotherapy (EP regimen) plus immunotherapy (atezolizumab/durvalumab), achieving a median survival of 12–13 months.

In recent years, immunotherapy has reshaped advanced NSCLC treatment, emerging as a pivotal first-line standard using PD-1/PD-L1 inhibitors as monotherapy or in chemotherapy combinations. For PD-L1 expression ≥50% tumors, pembrolizumab monotherapy yields 45% objective response rate (ORR), while KEYNOTE-189 combination regimens extend overall survival (OS) from 11.3 to 22 months. In SCLC, atezolizumab-plus-chemo regimens prolong median OS from 10.3 to 12.3 months. However, despite their transformative impact via PD-1/PD-L1 axis blockade, a substantial subset of patients develops primary or acquired resistance. To address therapeutic resistance, combination strategies integrating immune checkpoint inhibitors (ICIs) with radiotherapy, targeted agents (e.g., anti-angiogenic drugs), microbiome modulation, or metabolic interventions (such as JAK inhibitors) exhibit synergistic efficacy. These approaches collectively remodel the tumor microenvironment (TME), enhance antigen presentation, and reverse T-cell exhaustion, thereby overcoming key resistance barriers.

The tumor microenvironment of lung cancer constitutes a dynamic ecosystem composed of diverse immune cells, stromal components, extracellular matrix, and bioactive molecules. As a critical niche supporting tumor growth and survival, the TME exerts profound influences on immune cell function and activity due to its intricate composition and unique physicochemical properties. These characteristics are closely associated with lung cancer progression and critically shape the efficacy of immunotherapeutic interventions. Central to this interplay is the TME’s metabolic landscape, which serves as a key regulator of immune cell functionality. Tumor and stromal cells undergo metabolic reprogramming, leading to the accumulation of metabolites that directly suppress anti-tumor immunity; concurrently, immune cells within the TME adapt metabolically, and their dynamic crosstalk with cancer cells modulates immune evasion and responses to immunotherapy. To sustain survival and proliferation in this hostile microenvironment—marked by hypoxia and nutrient scarcity—tumor cells rely on such metabolic rewiring [2]. This adaptation not only drives tumor growth but also remodels immune responses within the TME [3], a niche pivotal to tumor initiation, progression, invasion, and metastasis [4].

**Figure 1:**
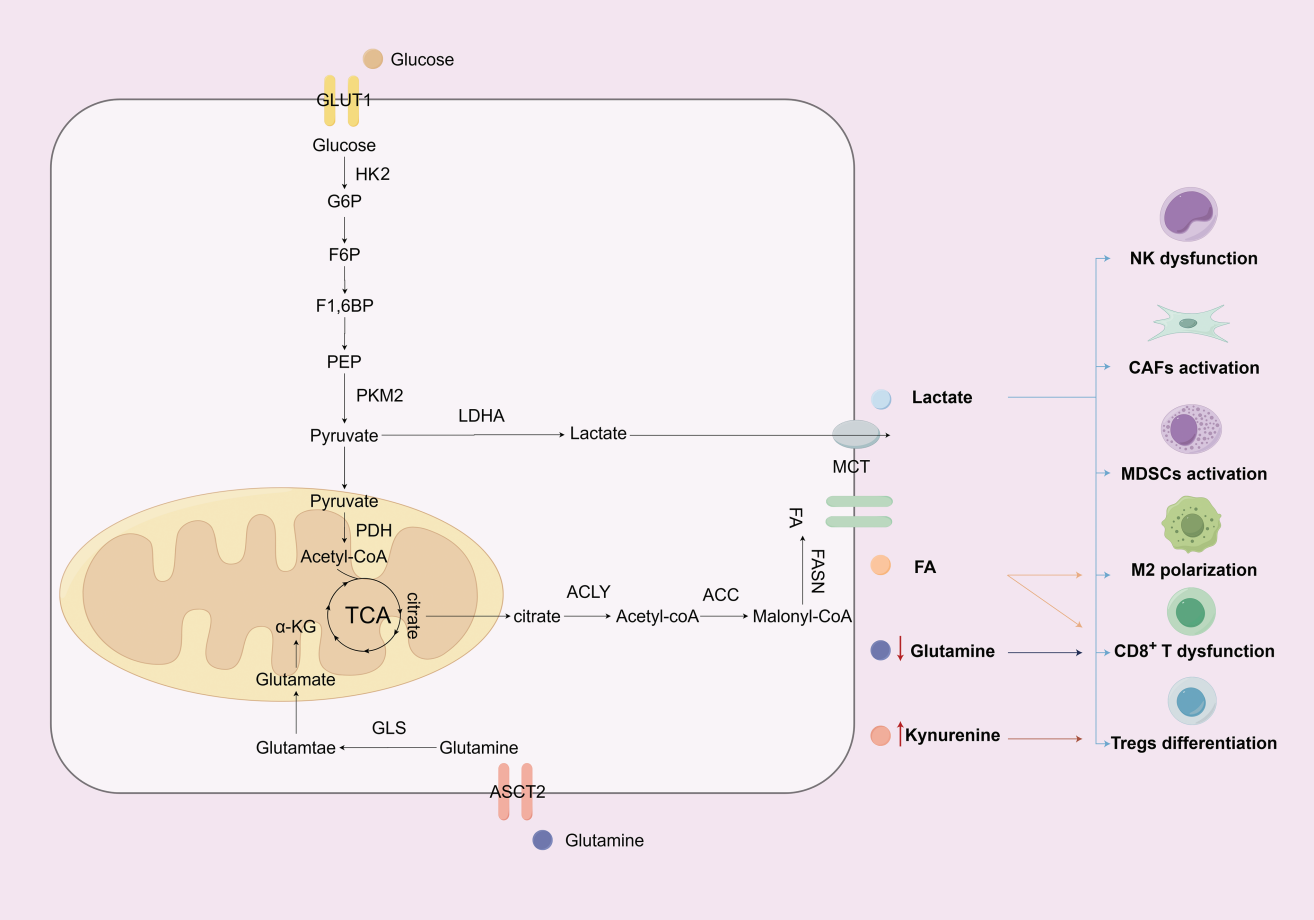
The impact of glucose, amino acid, and fatty acid metabolic reprogramming in lung cancer cells on the TME (Figure created with figdraw). This figure shows that lung cancer cells drive immune evasion through metabolic reprogramming and TME modulation. They enhance glycolysis via key molecules (GLUT1, HK2, PKM2, LDHA), export lactate to acidify the microenvironment—suppressing CD8^+^ T/NK cells while promoting M2 macrophages, CAFs, and MDSCs. In amino acid metabolism, ASCT2 uptakes glutamine for anabolism; its depletion and kynurenine (tryptophan metabolite) suppress CD8^+^ T cells and promote Tregs. For fatty acids, upregulated ACC/FASN boost synthesis, aiding proliferation and M2 activation while suppressing CD8^+^ T cells. These pathways synergize to drive immune evasion. **Note: Abbs.:** GLUT1, Glucose Transporter 1; HK2, Hexokinase 2; G6P, Glucose-6-Phosphate; F6P, Fructose-6-Phosphate; F1,6BP, Fructose-1,6-Bisphosphate; PEP, Phosphoenolpyruvate; PKM2, Pyruvate Kinase M2; LDHA, Lactate Dehydrogenase A; PDH, Pyruvate Dehydrogenase; TCA, Tricarboxylic Acid Cycle; α-KG, α-ketoglutarate; GLS, Glutaminase; ASCT2, Alanine-Serine-Cysteine Transporter 2; MCT, Monocarboxylate Transporter; FASN, Fatty Acid Synthase; ACLY, ATP-Citrate Lyase; ACC, Acetyl-CoA Carboxylase;NK, Natural killer cells; CAFs, Cancer-associated fibroblasts; MDSCs, Myeloid-derived suppressor cells; Tregs, Regulatory T cells.

Emerging evidence links therapeutic resistance to three interconnected factors: the metabolic state of tumor cells, metabolic alterations in immune populations, and the TME’s complex regulatory network [5,6]. Against this backdrop, investigating the crosstalk between lung cancer metabolism and immune regulation is critically important for elucidating the pathogenesis, optimizing therapeutic strategies, and improving patient survival (Fig. 1). This review aims to elucidate the metabolic-immune crosstalk in lung cancer by summarizing advances in how metabolic reprogramming drives immune suppression, critically evaluating emerging metabolic-targeted therapies, and providing a theoretical framework for precision immunotherapy combinations.

## 2 Metabolic Reprogramming of Lung Cancer Cells

### 2.1 Glucose Metabolism in Lung Cancer

Glucose metabolism underpins cell proliferation and the establishment of terminal cellular states [7]. Tumor cells exhibit significant glucose metabolic reprogramming, marked by enhanced glucose uptake compared to normal cells and preferential redirection of glycolytic pyruvate to lactate production even under oxygen-replete conditions, a distinctive phenomenon referred to as the “Warburg effect” [8]. Although this glycolytic pathway results in inefficient ATP production due to lactate generation, it compensates for increased energy demands by significantly accelerating glycolytic flux, thereby facilitating rapid tumor cell proliferation and migration [9,10]. The glycolytic pathway comprises ten enzymatic reactions, among which the steps mediated by hexokinase (HK), phosphofructokinase (PFK), and pyruvate kinase (PK) function as irreversible rate-limiting nodes that are druggable targets for effectively blocking glycolysis, thereby reducing energy supply and suppressing tumor cell proliferation [11].

Glucose uptake into cells via glucose transporter proteins (GLUTs) is a prerequisite for glycolysis. In lung cancer, the upregulated expression of GLUT1 facilitates glucose uptake, thereby supplying sufficient energy for rapid tumor cell proliferation [12,13]. Jang et al. [14] observed that tobacco-induced hyperglycemia drives a metabolic-immune axis centered on GLUT1 in lung adenocarcinoma (LUAD), where GLUT1 overexpression enhances glucose uptake in both tumor cells and tumor-associated macrophages (TAMs), directly inducing glycolytic metabolic reprogramming. This metabolic dysregulation promotes TAMs to secrete insulin-like growth factor 2 (IGF2) in a paracrine manner, activating the insulin receptor (IR) signaling pathway in lung cancer cells. The activated IR undergoes nuclear translocation and forms a complex with nucleophosmin (NPM1), thereby upregulating PD-L1 expression. This GLUT1-dominated axis not only remodels the TME—notably by increasing M2-like TAM infiltration and suppressing T cell function—but also synergistically promotes immune evasion and tumor progression, highlighting GLUT’s pivotal role in linking glycolysis to TME regulation in LUAD. Zhou et al. [15] found that GLUT1 is overexpressed at both transcriptional and translational levels in LUAD, and its high expression is positively correlated with advanced clinical stage, poor differentiation, and lymph node metastasis. In LUAD patients, higher GLUT1 expression predicts poorer prognosis, which is further exacerbated when combined with Epidermal Growth Factor Receptor (EGFR) 19DEL or L858R mutations. Mechanistically, GLUT1 directly interacts with phospho-EGFR (p-EGFR) and inhibits EGFR protein degradation via the ubiquitin-proteasome pathway. Notably, GLUT1 inhibitors can enhance the sensitivity of LUAD cells to gefitinib. These findings suggest that targeting GLUT1 alone or in combination with EGFR-TKIs may represent a promising therapeutic strategy for LUAD.

Glucose is phosphorylated to glucose-6-phosphate (G6P) by hexokinase 2 (HK2), an enzyme frequently overexpressed in multiple malignancies [16]. HK2 binds to the voltage-dependent anion channel (VDAC) on the outer mitochondrial membrane, and this localization not only prevents feedback inhibition by G6P, but also enhances glycolytic flux [17]. HK2 promotes stemness properties in tumor cells, and its overexpression in NSCLC is related to poor patient prognosis [18,19]. Daurisoline, a bis-benzylisoquinoline alkaloid isolated from the traditional Chinese herb *Menispermum dauricum*, was demonstrated by Tan et al. [20] to suppress glycolysis and remodel the TME in lung cancer through modulation of the AKT-HK2 axis. Mechanistically, daurisoline directly binds to AKT, inhibiting its phosphorylation at Ser473; this reduction in AKT activity subsequently decreases GSK3β phosphorylation at Ser9, which enhances the ubiquitination and proteasomal degradation of c-Myc—a critical transcription factor that drives HK2 expression. Li et al. [21] proved that the circular RNA circRUNX1 acts as a molecular sponge to competitively bind miR-145, thereby relieving the post-transcriptional repression of HK2 mRNA by miR-145 and significantly upregulating HK2 protein expression. The elevated HK2 expression potently enhances the Warburg effect—characterized by increased glucose uptake, augmented lactate production, and elevated ATP generation—within tumor cells. The excessive lactate accumulated in the TME leads to microenvironmental acidification, which in turn promotes the infiltration and proliferation of regulatory T cells (Tregs) while suppressing the function of cytotoxic T cells, ultimately driving immune evasion in NSCLC. The findings highlight the critical role of the circRUNX1/miR-145/HK2 pathway in linking metabolic reprogramming to immune escape in NSCLC, providing a rationale for targeting this axis in cancer therapy.

Pyruvate kinase (PK) catalyzes the transformation of phosphoenolpyruvate and ADP into pyruvate and ATP [22]. During tumorigenesis, PKM1 or PKML/R gradually declines, while PKM2 is significantly upregulated, making PKM2 the tumor-specific isoform of pyruvate kinase [23]. Long et al. [24] demonstrated that PKM2 exhibits high expression in LUAD. Its elevated expression correlates with tumor invasiveness, positioning PKM2 as an independent prognostic marker that may guide therapeutic targeting to improve lung cancer outcomes. In the research conducted by Markowitz et al. [25], PKM2 regulates the differentiation of CD8^+^ T cells and their responsiveness to PD-1 checkpoint blockade. That is, by inhibiting glycolysis through PKM2, the activity of the pentose phosphate pathway (PPP) increases, leading to the enrichment of CD8^+^ T cells phenotype with TCF1^high^ progenitor exhausted-like population, and enhancing the responsiveness to PD-1 blockade *in vivo*.

Hypoxia-inducible factor-1α (HIF-1α) modulates the expression of multiple critical glycolytic enzymes [26]. HIF-1α upregulates glycolytic enzyme activity by 90%, generates excessive lactate effluxing into the TME, induces metabolic competition with T cells, and ultimately suppresses lymphocyte antitumor function to promote immune evasion [27]. In patients with NSCLC, HIF-1α-positive tumor expression correlates with lower overall survival compared to HIF-1α-negative expression [28]. During NSCLC progression, aldolase A (ALDOA) expression positively correlates with nuclear HIF-1α levels. ALDOA overexpression enhances lactate secretion, which subsequently inhibits PHD enzymatic activities and contributes to HIF-1α protein stabilization [29], while reactive oxygen species (ROS) also stabilize HIF-1α by inhibiting PHD [30]. The tumor-suppressive effects of PHD2 on NSCLC proliferation, metabolic regulation, and ROS induction entirely rely on its enzymatic activity, as loss of activity fully abolishes these functions [31].

Notably, tumor cell metabolism demonstrates significant heterogeneity rather than a uniform pattern. Tumor cells adjacent to blood vessels predominantly utilize oxidative phosphorylation (OXPHOS) to generate energy, while hypoxic regions rely on glycolysis for glucose metabolism. This spatial metabolic heterogeneity fosters a symbiotic network, enabling tumor cells to adapt to the fluctuating hypoxic environment [32]. Neuroendocrine (NE) cells in small cell lung cancer (SCLC) exhibit electrophysiological excitability, with their action potential discharges contributing to tumor progression. This electrical activity not only promotes tumor expansion but also results in increased ATP demand and a strong reliance on OXPHOS [33]. NSCLC cells with CIP2A overexpression exhibit metabolic reprogramming characterized by increased OXPHOS and suppressed aerobic glycolysis [34]. Additionally, apoptosis-inducing factor (AIF) promotes tumor cell proliferation in Kras^G12D^-driven lung cancer by regulating OXPHOS, and high AIF expression correlates with unfavorable patient outcomes [35].

### 2.2 Amino Acid Metabolism in Lung Cancer

Glutamine (GLN), with its uptake and metabolic demand significantly increased in tumor cells under malignant contexts, acts as a conditionally essential amino acid under tumor metabolic reprogramming [36]. Lung cancer cells achieve efficient glutamine uptake through high expression of the alanine-serine-cysteine transporter-2 (ASCT2) [37]. Cancer cells exhibit elevated GLN consumption compared to other amino acids, promoting their rapid proliferation [36,38]. Once inside cells, glutaminase (GLS) catalyzes the conversion of GLN to glutamate, which is then transformed into α-ketoglutarate (α-KG) that enters the TCA cycle. Among GLS isoforms, GLS1 overexpression in various cancers promotes tumor stage progression [39]. GLS1 produces two splice variants—glutaminase C (GAC) and kidney-type glutaminase (KGA)—through alternative pre-mRNA splicing, both of which exhibit differential regulatory activities [40]. GAC is upregulated in lung cancer, a phenomenon closely linked to GLN metabolic reprogramming [41]. Increasing GLN levels in the TME may further enhance T cell activation and proliferation, thereby strengthening antitumor immunity [42].

Oncogene activation drives significant GLN dependence in lung cancer cells [43,44]. The transcription factor c-MYC, which regulates cell growth, proliferation, and metabolism, is frequently overexpressed in lung cancer and directly upregulates the key metabolic enzyme GLS1 [45,46]. c-MYC regulates GLN uptake and hydrolysis by modulating ASCT2 and GLS1 expression [47], while also regulating mitochondrial respiratory chain function and biogenesis, thereby influencing GLN metabolism [48]. In LUAD, the transcription factor TFDP1 transcriptionally activates the expression of SPC25 by binding to its promoter region, resulting in SPC25 overexpression [49]. This event markedly enhances the GLN metabolic pathway, which drives the synthesis and accumulation of Glutathione (GSH). Elevated GSH levels impair the antitumor immune function of natural killer (NK) cells through multiple mechanisms: suppressing the expression of activation receptors on NK cells, reducing cytotoxicity, and diminishing the secretion of key effector molecules such as perforin, granzyme B, and interferon-gamma (IFN-γ). Wu et al. [50] revealed that HHLA2 inhibits glutamine metabolism in CD8^+^ T cells via the KIR3DL3 receptor, driving immune escape in EGFR-mutant LUAD. Blocking the HHLA2-KIR3DL3 axis restores T cell function and exerts synergistic anti-tumor effects when combined with EGFR-TKIs. Thus, HHLA2 antibodies alone or in combination with EGFR-TKIs may serve as a novel therapeutic option for EGFR-mutant lung cancer patients, particularly those with immunotherapy resistance.

Despite the high uptake of glucose and glutamine by cancer cells, these nutrients often remain insufficient to fully meet the biosynthetic demands. Instead, serine and other similar amino acids provide a significant portion of the carbon and nitrogen needed for cancer cell survival [51]. Under serine-sufficient conditions, PKM2 is activated by serine to maintain efficient glycolysis; however, when serine is limited, reduced PKM2 activity redirects glycolytic intermediates toward the serine synthesis pathway (SSP) [52]. Within this pathway, 3-phosphoglycerate (3-PG), a glycolytic intermediate, is converted into 3-phosphohydroxypyruvate via phosphoglycerate dehydrogenase (PHGDH). It is subsequently transformed into 3-phosphoserine (3-PS) and α-KG via phosphoserine aminotransferase (PSAT1). Ultimately, 3-PS is transformed into serine via phosphoserine phosphatase (PSPH) [53,54]. In lung adenocarcinoma, high PHGDH protein expression and elevated mRNA levels of key SSP enzymes are linked to poor overall survival [53]. PSAT1 upregulation in NSCLC enhances cancer cell proliferation, metastasis, and chemotherapy resistance, leading to adverse outcomes [54].

### 2.3 Lipid Metabolism in Lung Cancer

Cancer cells also undergo lipid metabolic reprogramming to acquire energy reserves for rapid proliferation and membrane synthesis substrates [55]. Non-malignant cells preferentially utilize exogenous fatty acids to meet metabolic demands, whereas malignant cells activate *de novo* synthesis pathways through metabolic reprogramming to sustain accelerated proliferation and heightened biosynthetic requirements [56]. Lipids can enter cells via CD36 or passive diffusion, with fatty acid-binding proteins (FABPs) facilitating their uptake [57]. *De novo* fatty acid synthesis initiates with the transport of citrate from mitochondria to the cytosol via the citrate-pyruvate cycle; subsequently, citrate is cleaved by ATP-citrate lyase (ACLY) into cytosolic acetyl-CoA and oxaloacetate; acetyl-CoA carboxylase (ACC) then catalyzes the carboxylation of cytosolic acetyl-CoA to generate malonyl-CoA; finally, fatty acid synthase (FASN) condenses acetyl-CoA and malonyl-CoA into 16-carbon palmitate in the presence of NADPH. Through the elongase system, the resulting palmitate is extended to form long-chain fatty acids or is desaturated via stearoyl-CoA desaturase-1 (SCD1) [58–60]. In human lung cancer tissue, aberrantly high expression levels of ACLY and FASN drive tumor progression by promoting lipogenesis, which is significantly linked to unfavorable prognosis in patients [61]. Elevated SCD1 levels in lung cancer are closely associated with enhanced cancer cell invasiveness, poorer prognosis, and increased chemoresistance [62]. In NSCLC, EGFR-TKI-resistant cells exhibit significant lipid droplet accumulation and upregulated SCD1 expression compared to drug-sensitive cells, with this phenotype driving the maintenance of drug resistance [63]. Fatty acid oxidation (FAO) requires carnitine palmitoyltransferase1 (CPT1) to activate long-chain fatty acids [64,65]. CPT1C shows elevated expression in lung cancer, where it promotes FAO to increase ATP and ROS generation for metabolic stress adaptation [66], and enhances the chemoresistance of NSCLC cells to cisplatin [67]. Arachidonic acid (AA), a long-chain polyunsaturated fatty acid, plays multifaceted roles in tumor progression, including promoting cell cycle progression, enhancing proliferation, and facilitating metastasis. Additionally, AA contributes to malignant behavior by modulating the TME. Studies [68] have shown that PLA2G10-mediated AA secretion promotes LUAD progression by suppressing CD4^+^ T cell activation, with minimal impact on the proliferation and migration of tumor cells themselves. Mechanistically, PLA2G10 elevates AA levels in the TME, which inhibits the phosphorylation of LCK (Y394) and ZAP70 (Y391), thereby suppressing CD4^+^ T cell activation and fostering immunosuppression. Combination therapy with PLA2G10 inhibitors and anti-PD-1 antibodies has demonstrated efficacy in lung cancer treatment. These findings suggest that AA-centered metabolic profiling could serve as a prognostic indicator for LUAD and identify AA metabolism as a potential therapeutic target.

### 2.4 Epigenetic Regulation

The epigenetic regulation of lung cancer is a sophisticated and dynamically evolving process [69,70], mainly involving DNA methylation, histone modification and non-coding RNA (ncRNA). Dysregulation of epigenetic mechanisms can drive tumor initiation and progression through metabolic reprogramming in cancer cells.

#### 2.4.1 DNA Methylation and Demethylation

DNA methylation requires the involvement of DNA methyltransferases (DNMTs) [71], mainly including DNMT1, DNMT3A and DNMT3B. Aberrantly elevated expression of DNMTs is associated with lung cancer progression [72]. Among them, DNMT1 overexpression induces the silencing of tumor suppressor genes by promoting promoter hypermethylation, disrupts the synergistic regulatory network of the p53/Sp1 signaling pathway, and eventually facilitates lung cancer initiation, development, and poor patient prognosis [73]. Study [74] shows that DNMT1 inhibition reverses the hypermetabolic state of cancer cells by reducing glycolysis and antioxidant capacity. Mechanistically, DNMT1 drives metabolic reprogramming through epigenetic regulation of the SCARA5/AOX1 axis, offering a new therapeutic target for NSCLC. Meanwhile, the TET (Ten-eleven translocation) family—key mediators of DNA demethylation—dynamically reverses DNA methylation via stepwise oxidation of 5-methylcytosine (5mC) by its core members TET1, TET2, and TET3. Rahim et al. [75] identified TET1 as a critical tumor suppressor in LC, acting through dual mechanisms of epigenetic remodeling and immune regulation. Specifically, TET1 loss promotes tumor progression by upregulating translation- and metabolism-related genes, thereby enhancing metabolic and biosynthetic pathways. In contrast, TET1 overexpression drives epigenetic reprogramming via catalytic DNA demethylation and strengthens antitumor immunity by activating the TNFα/NF-κB signaling pathway.

#### 2.4.2 Histone Modifications

Histone modifications encompass multiple forms, such as methylation, acetylation, and lactylation. Histone methylation occurs on the nitrogen atoms of side chains in lysine and arginine residues [76]. Mutations in the histone methyltransferase KMT2D are common in lung cancer. Loss of KMT2D impairs the super-enhancer of PER2, leading to upregulated glycolysis-related gene expression and promoting lung tumorigenesis [77]. SETD1A, a histone methyltransferase, is overexpressed in lung cancer. It activates the transcription of oncogenes such as MYC by catalyzing the H3K4me3 modification, thereby accelerating the proliferation of lung tumors [78]. Compared with adjacent normal lung tissue, the demethylase KDM2 is upregulated in tumor tissue from NSCLC patients [79]. Histone deacetylases (HDACs) are crucial in maintaining histone acetylation homeostasis [80]. Inhibition of HDACs reverses the silencing of tumor suppressor genes, induces cell cycle arrest, or triggers apoptosis [81]. HDAC1 overexpression in NSCLC drives TME remodeling via hypoxia-induced acidosis and enhanced lipogenesis, resulting in suppression of antitumor T cell immunity [82]. HDAC4 activates the glutaminase activity of GAC through deacetylation, thereby promoting glutamine metabolic reprogramming [83]. Lactylation modulates the expression of metabolic genes, in turn changing cellular energy metabolism pathways [84]. Lactate, a metabolite, regulates glycolysis and cell proliferation in NSCLC by mediating the expression of pertinent genes through histone lactylation [85].

NcRNAs drive metabolic reprogramming in cancer to facilitate tumor cell energy supply, biosynthesis, and microenvironmental adaptation, thereby promoting malignant tumor progression [86]. miR-144 is downregulated in lung cancer, and its loss of function activates glycolytic pathways by targeting GLUT1, thereby supplying energy for tumor cell proliferation [87]. miR-21 drives cell growth and migration in NSCLC by triggering the CD36-mediated fatty acid metabolic pathway [88]. lncRNAs participate in cell cycle regulation, gene regulation, immune response, and tumor metabolism [89]. lnc-IGFBP4-1 upregulates HK2, PDK1, and LDHA, thereby promoting lung cancer cell proliferation [90]. Accumulating evidence indicates that certain lncRNAs function as diagnostic staging biomarkers, prognostic predictors, and therapeutic targets during tumorigenesis and progression [91]. circRNAs modulate gene expression across the epigenetic and transcriptional levels [92,93]. The circ-PITX1/miR-1248/CCND2 axis modulates glycolysis and glutamine metabolism in NSCLC [94]. The inherent structural stability of circRNAs makes them ideal candidate biomarkers for cancer diagnosis [95]. circRNA_001846 is upregulated in NSCLC patients, and its serum levels may serve as a promising early diagnostic biomarker [96]. Furthermore, plasma circFARSA also demonstrates potential as a biomarker for NSCLC, providing research insights for the application of circRNAs in non-invasive cancer diagnosis [97].

## 3 Immune Metabolic Regulation and Immunosuppressive Network in the Lung Cancer Tumor Microenvironment

The tumor microenvironment, serving as a critical niche for tumor cell growth and survival, is a complex and dynamic ecosystem comprising tumor cells, immune cells, stromal cells, extracellular matrix (ECM) components, and various bioactive molecules. Within this ecosystem, metabolic and immune dysregulation plays a pivotal role in driving lung cancer progression and therapy resistance.

### 3.1 Metabolic Reprogramming of Immune Cells

#### 3.1.1 T Cells

Unlike cancer cells, the metabolic reprogramming of T cells requires co-stimulatory molecules and is initiated by TCR-mediated antigen recognition. This metabolic shift provides essential substrates and energy for T cells effector functions [98]. Naive T cells primarily rely on OXPHOS for energy production, whereas upon TCR-mediated activation, their metabolic phenotype shifts toward glycolysis to meet the heightened energy demands [99]. GLUT1 gene knockout or treatment with its inhibitors significantly impairs CD4^+^ T cell proliferation, effector differentiation, and inflammatory cytokine secretion [100]. Glucose deprivation in the TME restricts aerobic glycolysis in T cells, thereby impairing their antitumor capacity [101]. T cell activation is accompanied by significantly enhanced glycolysis and glutamine metabolism, whereas changes in FAO vary with T cell subsets and the microenvironment [102]. ASCT2 expression is low in naive or resting T cells but is markedly upregulated within hours of activation [103]. Intracellular L-arginine not only bolsters the survival capacity of both human and murine T cells but also promotes the differentiation of central memory-like T (Tcm) cells. In murine models, Tcm cells outperform effector memory T cells in tumor eradication [104]. Excessive lipid accumulation, commonly observed in CD8^+^ TILs, induces functional impairment and exhaustion, while in response to lipid-rich TME and nutrient stress, these cells upregulate lipid transporters to acquire lipids as alternative energy sources [105]. In the hypoxic and glucose-deprived TME, CD8^+^ TILs partially preserve their effector functions through enhanced PPAR-α signaling and fatty acid metabolism. This is reflected by the upregulated expression of lipid metabolism-related genes and accumulated metabolic intermediates [106].

#### 3.1.2 Dendritic Cells

Dendritic cells (DCs), the most potent antigen-presenting cells in the immune system, play pivotal roles in anti-tumor immunity and the TME. They are primarily categorized into two major subtypes: conventional dendritic cells (cDCs) and plasmacytoid dendritic cells (pDCs), with cDCs further subdivided into distinct functional subsets. Characterized by XCR1 and Clec9A expression, cDC1 specializes in cross-presenting antigens via the MHC-I pathway to recruit and activate CD8^+^ T cells, while cDC2—marked by CD11b and CD172a—primarily enhances the functions of CD4^+^ helper T cell 17 (Th17) and Th1 cells. Lobel et al. [107] found that GLN regulates the survival, proliferation, and differentiation of type 1 conventional dendritic cells (cDC1s) via the mTORC1 signaling pathway. cDC1s are highly dependent on GLN levels, and its deficiency suppresses mTOR signaling, triggering increased apoptosis and impaired differentiation—effects less pronounced in cDC2s. This leads to reduced cDC1 numbers and frequency within tumors, impairing antigen cross-presentation and T cell activation, thereby promoting immune evasion. Additionally, glycolysis serves as the metabolic foundation for DCs in anti-tumor immunity. Glycolysis-deficient DCs fail to suppress tumor growth effectively, accompanied by reduced infiltration and effector functions (e.g., IFN-γ and granzyme B production) of tumor-infiltrating CD4^+^ and CD8^+^ T cells. By elevating intracellular ATP levels in DCs—essential for STING phosphorylation—glycolysis drives STING signaling activation. STING activation further upregulates HIF-1α, which enhances accumulation of glycolytic key enzymes (e.g., HK2 and PKM2), forming a positive feedback loop. This metabolic reprogramming, particularly glycolysis-driven, acts as a DC activation driver: it promotes type I interferon responses and T cell activation via ATP-STING signaling, while STING reinforces glycolysis through HIF-1α [108]. Clinical studies confirm the universality of this axis in NSCLC, offering a novel strategy for DC metabolism-targeted immunotherapy.

#### 3.1.3 Natural Killer Cells

Natural killer (NK) cells, as innate immune lymphocytes, have garnered significant research attention for their metabolic reprogramming during antitumor immune responses. The metabolic stress imposed by the TME critically governs NK cells survival, proliferation, cytokine production, cytotoxic capacity, and ultimately, their efficacy in cancer immunotherapy. In the TME of lung adenocarcinoma, significant downregulation of FABP4 and SPON2 disrupts lipid homeostasis in NK cells, reducing key effector molecule expression and markedly impairing cytotoxic function [109]. *In vitro* experiments confirmed that this metabolic reprogramming arrests NK cells in an immature state, compromising antitumor immunity. Huang et al. [49] revealed that TFDP1 transcriptionally activates SPC25 to upregulate glutamine metabolism, thereby suppressing NK cells antitumor immunity. Mechanistically, SPC25 overexpression enhances glutamine uptake and metabolite production (e.g., glutamate, α-ketoglutarate), resulting in reduced NK cells cytotoxicity, decreased expression of activating receptors, and diminished secretion of perforin, granzyme B, and IFN-γ. Knockdown of TFDP1 reverses this effect, whereas SPC25 overexpression reinstates NK cells suppression, confirming metabolic reprogramming as the core immunosuppressive mechanism. This offers a novel strategy to enhance immunotherapy by targeting the TFDP1/SPC25 axis.

### 3.2 Immune Suppressive Network Constructed by the Tumor Microenvironment

**Figure 2:**
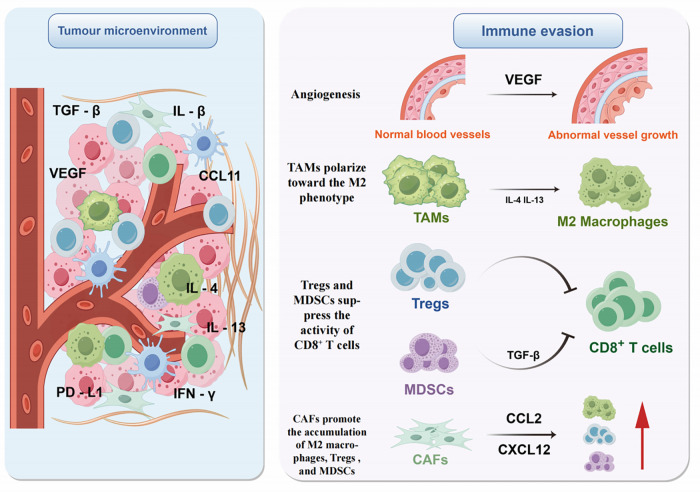
Immune evasion mediated by the tumor microenvironment (Figure created with figdraw). This figure illustrates key mechanisms by which the TME mediates immune evasion. Enriched with cytokines (e.g., TGF-β, VEGF, IL-1β) and diverse immune cells, the TME drives suppression via multiple pathways: VEGF promotes aberrant angiogenesis; IL-4/IL-13 polarizes TAMs to the M2 phenotype; Tregs and MDSCs inhibit CD8^+^ T cells via TGF-β; and CAFs recruit M2 macrophages, Tregs, and MDSCs by secreting CCL2/CXCL12. These mechanisms synergize to promote immune evasion. **Note: Abbs.:** TGF-β, Transforming Growth Factor-beta; IL-1β, Interleukin-1 beta; IL-4, Interleukin-4; IL-13, Interleukin-13; VEGF, Vascular Endothelial Growth Factor; CCL11, C-C motif chemokine ligand 11; PD-L1, Programmed Cell Death-Ligand 1; IFN-γ, Interferon-gamma; TAMs, Tumor-Associated Macrophages; Tregs, Regulatory T cells; MDSCs, Myeloid-derived suppressor cells; CAFs, Cancer-associated fibroblasts.

The immune suppression in the TME is not driven by a single pathway but rather constitutes a complex network resulting from the concerted action of multiple mechanisms, including metabolic competition, secretion of inhibitory factors, immune cell polarization, and immune checkpoint activation [110] (Fig. 2).

The hypoglycemic TME suppresses the PI3K-Akt-mTOR signaling pathway, downregulating glycolysis in CD4^+^ T cells and driving their metabolic reprogramming toward FAO and OXPHOS to meet biosynthetic demands for energy [111]. In LUAD patients, IL-38 overexpression reduces CD8^+^ TILs and suppresses antitumor immunity [112]. Tumor cells promote Tregs differentiation and suppress antitumor immune responses by generating kynurenine through the IDO-mediated tryptophan metabolic pathway [113]. Tumor-derived factors drive TAMs to secrete various cytokines, suppressing T cell-mediated immune responses and exacerbating the formation and maintenance of an immunosuppressive TME [114]. Lactate downregulates ATP6V0d2 expression in TAMs to promote HIF-2α-mediated tumor progression. Tumor tissue analysis from 35 lung cancer patients revealed a negative correlation between ATP6V0d2 and HIF-2α expression in TAMs. Moreover, high ATP6V0d2 expression correlated with longer survival, while high HIF-2α was associated with shorter survival [115]. Lung cancer cell-secreted macrophage colony-stimulating factor drives TAMs to M2 polarization by upregulating FASN expression and activating PPARβ/γ, thereby stimulating tumor angiogenesis and facilitating cancer cell invasion into the stroma [116]. Furthermore, by lowering amino acid levels and secreting lactate, M2 macrophages dampen the antitumor effector function of NK cells and T cells [117].

In the hypoxic TME, HIF-1α activation in NK cells upregulates HIF-1α-dependent genes that promote angiogenesis, such as VEGF and TGF-β [118]. Knockout of HIF-1α in NK cells enhances their antitumor response [119]. In the TME of KRAS-driven lung cancer, high TGF-β concentrations induce aberrant overexpression of FBP1 in NK cells, suppressing glycolysis and reducing cellular activity, ultimately compromising their antitumor function [120].

The TME promotes tumor immune evasion through PD-1/PD-L1 pathway activation [121]. In NSCLC, TGF-β1 reduces DNMT1 activity, leading to promoter demethylation of the PD-L1 gene and subsequent upregulation of PD-L1 expression [122]. Compared with adjacent non-tumor tissue, IL-1β expression is elevated in LUAD and contributes to cancer cell survival by upregulating PD-L1 expression [123]. Group 2 innate lymphoid cells (ILC2s) promote IL-4/IL-13 secretion through upregulated PD-1 signaling activity, driving M2 macrophage polarization and thereby enhancing the immunosuppressive TME [124]. VEGF-A enhances PD-1 expression on CD8^+^ T cells by binding to VEGFR2 on their cell surface [125]. Osteopontin (OPN) expressed by TAMs upregulates PD-L1 expression in NSCLC, thereby exacerbating tumor progression [126].

TAMs are functionally categorized into M1 and M2 subtypes, with M1 macrophages serving as critical effectors of antitumor immunity in early-stage cancer by employing multiple mechanisms to inhibit tumor growth and progression [127]. However, prolonged exposure to the TME, under the influence of external stimuli, drives TAMs to preferentially polarize to the M2 phenotype [128]. M2 macrophages, possessing anti-inflammatory and protumor properties, establish an immunosuppressive microenvironment by secreting anti-inflammatory cytokines. These cytokines simultaneously foster tumor angiogenesis while suppressing antitumor immunity [129–131]. M1 macrophages primarily rely on glycolysis and the pentose phosphate pathway to generate energy, whereas M2 macrophages meet their metabolic demands by enhancing FAO and OXPHOS [132]. Th1 cells drive M1 macrophage polarization by secreting IFN-γ, whereas Th2 cells promote M2 polarization by secreting IL-4 and IL-13 [133]. TAMs take up tumor cell-derived fatty acids via CD36, which promotes their polarization to the M2 macrophage phenotype and establishes an immunosuppressive TME [134]. This lipid accumulation drives a metabolic shift from glycolysis to FAO to sustain energy production [135]. Glutaminolysis-derived α-KG fuels the TCA cycle, enhancing FAO in M2 macrophages and thereby epigenetically driving M2 gene reprogramming in a histone methylation-dependent manner [136]. Suppressing glutamine synthetase (GS) activity in M2 macrophages promotes their transition to the M1 phenotype and enhances cytotoxic T cell recruitment [137].

Myeloid-derived suppressor cells (MDSCs), originating in the bone marrow, accumulate gradually during tumor progression in tumor-bearing hosts and migrate to peripheral lymphoid organs and tumor sites [138,139]. NADPH oxidase 2 (NOX2) is expressed by MDSCs and produces ROS to support the immunosuppressive TME [140]. In lung cancer, MDSCs are significantly elevated, where they secrete abundant TGF-β to suppress the immune system, induce T cell dysfunction, and consequently facilitate tumor growth and metastatic dissemination [141]. MDSCs can also promote the metastasis of NSCLC by highly expressing chemokines such as CCL11 to activate AKT and ERK signaling pathways [142]. Table 1 summarizes the regulatory mechanisms of metabolic reprogramming in lung cancer and their impact on the immune microenvironment.

**Table 1:** Regulatory mechanisms of metabolic reprogramming in lung cancer and their impact on the immune microenvironment.

Metabolic Pathway	Key Molecules	Metabolic Regulatory Mechanism	Impact on Immune Microenvironment	Clinical Significance	References
Glucose Metabolism	GLUT1 HK2 PKM2 HIF-1α	GLUT1 upregulation enhances glucose uptake. HK2 binding to mitochondrial VDAC potentiates glycolysis. PKM2 overexpression augments glycolytic flux. HIF-1α activates glycolytic enzymes.	Hypoglycemic tumor microenvironment impairs PI3K-Akt-mTOR signaling pathway, impelling CD4^+^ T cells toward FAO/OXPHOS and compromising effector functions. Lactate accumulation suppresses T and NK cells cytotoxicity. HIF-1α induces PD-L1 upregulation and VEGF secretion.	GLUT1/HK2/PKM2 overexpression correlates with poor prognosis. HIF-1α positivity predicts reduced survival.	[12,17,18,19,23,24, 28,111,117,118,147]
Amino Acid Metabolism	ASCT2 GLS1 c-MYC	c-MYC upregulates ASCT2 and GLS1 to regulate glutamine uptake and catabolism. PHGDH/PSAT1 overexpression activates serine biosynthesis.	Glutamine deprivation inhibits T cell activation. IDO-mediated tryptophan catabolism to kynurenine expands Tregs. Serine pathway activation depletes metabolic intermediates, disrupting immune cell homeostasis.	High GLS1 expression associates with advanced staging and poor outcomes. PHGDH/PSAT1 elevation predicts shortened survival.	[39,42,47,53,54,113]
Lipid Metabolism	ACLY FASN SCD1 CPT1	ACLY/FASN overexpression drives *de novo* lipogenesis. SCD1 upregulation promotes monounsaturated FA synthesis. CPT1C elevation enhances FAO and ROS generation.	Lipid overload induces CD8^+^ T cells exhaustion. FA uptake via CD36 drives TAMs polarization toward M2 phenotype.	FASN/ACLY/SCD1/CPT1C overexpression correlates with adverse prognosis. SCD1 elevation confers cisplatin resistance in NSCLC.	[61,62,66,105,134]

**Note: Abbs.:** GLUT1, Glucose Transporter 1; HK2, Hexokinase 2; PKM2, Pyruvate Kinase M2; HIF-1α, Hypoxia-Inducible Factor 1 Alpha; VDAC, Voltage-Dependent Anion Channel; OXPHOS, Oxidative Phosphorylation; FAO, Fatty Acid Oxidation; NK cells, Natural killer cells; PD-L1, Programmed Death-Ligand 1; VEGF, Vascular Endothelial Growth Factor; ASCT2, Alanine, Serine, Cysteine Transporter 2; GLS1, Glutaminase 1; c-MYC, Cellular myelocytomatosis viral oncogene homolog; PHGDH, Phosphoglycerate Dehydrogenase; PSAT1, Phosphoserine Aminotransferase 1; IDO, Indoleamine 2,3-Dioxygenase; Tregs, Regulatory T Cells; ACLY, ATP Citrate Lyase; FASN, Fatty Acid Synthase; SCD1, Stearoyl-CoA Desaturase 1; CPT1, Carnitine Palmitoyltransferase 1; CPT1C, Carnitine Palmitoyltransferase 1C; CD36, Cluster of differentiation 36; ROS, Reactive Oxygen Species.

Regulatory T cells (Tregs), as a key immunosuppressive subgroup, play a crucial role in tumor immune evasion, angiogenesis, and metastasis by suppressing anti-tumor immunity [143]. Elevated levels of Tregs in lung cancer are closely associated with poor cellular differentiation, advanced disease stages, and enhanced metastatic potential, and are significantly linked to resistance against immune checkpoint inhibitor (ICI) therapy [144]. Liang et al.’s [145] study found that tobacco carcinogens activate the α7nAChR receptor and transcription factor c-Jun, induce upregulation of IDO1 expression, promote the metabolism of tryptophan (Trp) to kynurenine (Kyn), lower the Trp/Kyn ratio. This metabolic reprogramming directly affects the immune cell metabolic microenvironment: Trp depletion inhibits effector T cell proliferation, Kyn accumulation acts as a ligand for the aryl hydrocarbon receptor (AhR), and Kyn activates AhR signaling, inducing the differentiation of naive CD4^+^ T cells into Tregs, thereby enhancing immunosuppressive function. Kratzmeier et al. [146] revealed that IFN-γ derived from LUAD cells activates CD8^+^ T cells to upregulate CCR5 chemokines (e.g., CCL3/CCL4), promoting the migration of CD4^+^Foxp3^+^ Tregs into the TME. Tregs suppress anti-tumor immunity via mechanisms including metabolic disruption (e.g., interfering with nutrient competition of effector T cells) and secretion of immunosuppressive cytokines (such as IL-10 and TGF-β). Notably, CCR5 antagonists can block this cascade, restore immune activity, and enhance the efficacy of ICI therapy in lung cancer.

Cancer-associated fibroblasts (CAFs) drive tumor progression by remodeling the extracellular matrix, regulating metabolism, and mediating crosstalk between cancer cells and immune cells [148]. CAFs can significantly elevate the proportion of M2 macrophages, Tregs, and MDSCs, ultimately facilitating tumor immune evasion [149]. In NSCLC, two CAF subtypes are linked to T cell exclusion: MYH11^+^αSMA^+^CAF and FAP^+^αSMA^+^CAF, which both localize around tumor parenchyma and promote T cell exclusion by depositing collagen fibers [150]. CAFs derived from NSCLC not only suppress the immune response of cytokine-activated NK cells [151], but also promote lung cancer cell migration via Four and a half LIM domain protein 2 (FHL2) expression, while FHL2 knockout significantly attenuates this pro-tumorigenic effect [152].

Tumor-related metabolites, such as tryptophan, lactic acid, adenosine, chemokines, and others, can also contribute to an immunosuppressive TME, thereby limiting the function of immune cells and the efficacy of immunotherapy [153,154]. Wu et al. [155] revealed that NDRG1-driven lactate accumulation induces histone H3K18 lactylation (H3K18la) in macrophages, upregulating immunosuppressive genes (e.g., CD163, PD-L1, IL-10). This process reinforces the M2 phenotype, establishes an immunosuppressive microenvironment, and ultimately drives lung adenocarcinoma progression. Ding et al. [156] discovered that chemoresistant tumor cells upregulate the expression of CD39 and CD73, promoting the conversion of ATP to adenosine (Ado), which leads to elevated Ado levels in the TME. This accumulated Ado activates the A2AR/PKA/mTORC signaling axis, driving metabolic reprogramming in TAMs and upregulating IDO1 expression. IDO1 catalyzes the breakdown of tryptophan, depleting this essential amino acid in the microenvironment, thereby suppressing the activation and proliferation of CD8^+^ T cells and facilitating immune escape. TAMs induced by resistant tumors inhibit T-cell cytotoxic function via the Ado–A2AR axis. Targeting this signaling node can reverse drug resistance and offers a novel strategy for combination immunotherapy.

## 4 Therapeutic Approaches Directed at Metabolism and Immunity in Lung Cancer

### 4.1 Metabolic Targets

Apoptin targets PKM2 to inhibit glycolysis in A549 lung cancer cells, while promoting autophagy and apoptosis through modulation of the PKM2/AMPK/mTOR pathway [157]. Pitavastatin inhibits NSCLC progression by blocking CD36 to disrupt the CD36/AKT/mTOR signaling pathway and reduce lipid accumulation [158]. ND-646, an allosteric inhibitor targeting ACC1, disrupts lung tumor growth by interfering with ACC1 protein subunit dimerization to block fatty acid synthesis [159]. Combined treatment with PKM2-IN-1 (a PKM2 inhibitor) and NCT-503 (a PHGDH inhibitor) synergistically decreases GLUT1 expression, effectively suppressing lung cancer cell proliferation [160]. The GLS inhibitor CB-839 not only enhances radiosensitivity in NSCLC patients by reducing intracellular GSH levels [161], but also synergizes with the targeted therapy selumetinib to potentiate antitumor activity against KRAS-mutant NSCLC [162].

### 4.2 Immune Targets

Numerous emerging immune checkpoint molecules, including TIGIT, LAG-3, PD-1, TGF-β, CD73, TIM-3, and B7-H4, are presently under investigation as prospective therapeutic targets in cancer. T cell immunoglobulin and ITIM domain (TIGIT) is primarily expressed on T cells and NK cells [163]. Luo et al. [164] demonstrated that IL-15 stimulation enhances effector function in CD8^+^ TILs while concurrently upregulating TIGIT expression; combinatorial treatment with IL-15 and TIGIT blockade synergistically enhances CD8^+^ TILs cytotoxicity and augments antitumor immunity in LUAD. Although anti-TIGIT agents have demonstrated promising potential in lung cancer therapy, their clinical translation currently faces significant challenges [165]. CD73, also known as ecto-5^′^-nucleotidase or NT5E, is a GPI-anchored cell surface ectonucleotidase that plays a pivotal role in purinergic signaling by catalyzing the hydrolysis of extracellular AMP into immunosuppressive adenosine, and is highly expressed on tumor and immune cells. As the final mediator of extracellular adenosine production across multiple pathways, inhibiting CD73 directly counters immunosuppressive adenosine accumulation-mediated immune evasion, establishing it as a key therapeutic target in cancer immunotherapy [166,167]. In senescent tumor cells induced by radiotherapy or chemotherapy, IL-6 secretion activates the JAK/STAT3 pathway in macrophages, driving CD73 transcriptional upregulation that catalyzes adenosine accumulation in the TME and suppresses CD8^+^ T cell proliferation along with Granzyme B and IFN-γ secretion [168]. CD73 inhibition abrogates adenosine production, restores antitumor immunity, and synergizes with anti-PD-1 antibodies to significantly suppress tumor growth and prolong survival; this combination therapy elevates CD8^+^IFN-γ^+^ T cell proportions and PD-1 upregulation, indicating T cell activation, thereby positioning CD73 targeting as a viable approach to overcome immunosuppression in aged lung cancer TMEs, particularly when combined with PD-1/PD-L1 inhibitors.

### 4.3 Targeting Gut Microbiota and Metabolites

Gut microbiota-derived metabolites act as immune modulators, exerting their effects on the TME and anti-tumor therapeutic outcomes through diverse mechanisms [169]. Key metabolites include short-chain fatty acids (SCFAs, e.g., acetate, propionate, butyrate), tryptophan derivatives (e.g., indole-3-lactate, indole-3-propionate), secondary bile acids (e.g., deoxycholic acid), polyamines (e.g., spermidine), and trimethylamine N-oxide (TMAO) [170]. These molecules have been shown to critically influence lung cancer progression and drug response [171]. Zhu et al. [172] discovered that in a lung cancer mouse model, oral gavage of gut *Akkermansia muciniphila* (Akk) enriched tumor microbiota with Akkermansia and other intestinal microbes, indicating gut-derived Akk migrates to lung tumor sites to reshape intratumoral microbiome composition and reprogram metabolic pathways for anticancer effects. This process downregulated key metabolites like lactate and glutamate in the tumor microenvironment, inhibiting glycolysis and glutamine metabolism, which modulated immune cell activity via changes in enzymes such as LDHA and GLS, thereby indirectly enhancing antitumor immune responses. Camu-camu (CC) is an Amazonian berry rich in phytochemicals, and castalagin is a polyphenol extracted from it. Research by Messaoudene et al. [173] revealed that oral castalagin supplementation reshapes gut microbiota composition—enriching beneficial bacteria like Ruminococcaceaeand Alistipes, enhancing microbial diversity, and increasing the ratio of CD8^+^ T cells to Tregs in the TME. It also induces metabolic shifts, including elevated taurine-conjugated bile acids, thereby boosting anti-tumor immunity. In NSCLC models, castalagin overcomes anti-PD-1 resistance and enhances the efficacy of ICIs via a microbe-dependent mechanism. The gut microbiota, host metabolism, and immune function constitute an intricate regulatory network. Therefore, by leveraging patient-specific data including microbiome profiling, metabolomic signatures, and tumor immune subtypes, personalized therapeutic strategies can be developed. These tailored combinatorial regimens may involve direct microbial manipulation, metabolite-targeted approaches, dietary interventions, strategic metabolite supplementation, or inhibition of detrimental metabolic pathways. The ultimate objective is to reprogram the immunosuppressive TME into an anticancer immune-permissive state.

### 4.4 Metabolic-Immune Combination Therapy

In NSCLC, KEAP1 mutations impair its ability to mediate ubiquitination-dependent PD-L1 degradation, resulting in PD-L1 accumulation and subsequent immune evasion. Thus, targeted restoration of KEAP1 activity to degrade PD-L1, combined with anti-PD-L1 therapy, synergistically enhances antitumor immunity [174]. DRP-104 targets the glutamine-dependent metabolic vulnerability of KEAP1-mutant lung cancer, inhibiting tumor growth and reversing T cell exhaustion. In combination with anti-PD-1 therapy, it exerts synergistic antitumor effects [175]. Targeted therapy against CPT1A combined with PD-1 antibody inhibits tumor growth, demonstrating superior efficacy compared with monotherapy [176]. HDAC inhibitors (e.g., entinostat) enhance the synergistic antitumor efficacy of anti-PD-1 therapy in murine lung cancer models by suppressing metabolism-linked immunosuppressive factors (e.g., arginase-1, iNOS) in MDSCs [177]. In NSCLC, YAP/TAZ transcriptionally upregulate PD-L1 expression to promote tumor immune evasion, while combined inhibition of YAP/TAZ and PD-1/PD-L1 synergistically boosts antitumor immune responses [178]. MK1775 inhibits the PI3K/AKT/mTOR pathway in tumor cells, reduces fatty acid oxidation in TAMs, and promotes CD8^+^ T cell infiltration, while its combination with anti-PD-1 therapy improves treatment response rates in lung adenocarcinoma patients with lipid metabolism abnormalities and overcomes immune resistance [179]. Anti-PD-1/PD-L1 therapy, when combined with metformin, enhances antitumor immune responses in peripheral immune cells from NSCLC patients [180]. In NSCLC tissue samples, PD-L1 expression correlates positively with HIF-1α levels, suggesting that combined targeting of PD-L1 and HIF-1α may represent a more effective precision therapeutic strategy for NSCLC [147].

Therefore, combining metabolic pathway targeting with immune checkpoint blockade represents a prospective therapeutic approach for lung cancer (Table 2).

**Table 2:** Targeted metabolic-immune therapeutic strategies.

Target/Pathway	Mechanism of Action	Combined Immunotherapy	Effect	Reference
KEAP1	KEAP1 mutations cause PD-L1 accumulation for immune evasion. DRP-104 targets KEAP1-mutant lung cancer glutamine-dependent metabolic vulnerability.	Anti-PD-1/PD-L1 antibody	Synergistically enhances antitumor immunity.	[174,175]
CPT1A	Inhibits key FAO enzyme CPT1A, reprogramming tumor metabolism.	Anti-PD-1 antibody	Suppresses tumor growth more effectively than monotherapy.	[176]
HDAC	HDAC inhibitors (e.g., entinostat) suppress immunosuppressive factors (e.g., Arg-1, iNOS) in MDSCs.	Anti-PD-1 antibody	Improves Tcell function and enhances response to immunotherapy.	[177]
YAP/TAZ	YAP/TAZ transcriptionally activate PD-L1 to enhance tumor immune escape.	Anti-PD-1/PD-L1 antibody	Restore Tcell activation, proliferation, and effector function.	[178]
PI3K/AKT/mTOR	MK1775 inhibits pathway, reduces FAO in TAMs, and promotes CD8^+^ T cell infiltration.	Anti-PD-1 antibody	Increases response rate in LUAD with dysregulated lipid metabolism and overcomes immunotherapy resistance.	[179]
AMPK	Metformin activates AMPK signaling.	Anti-PD-1/PD-L1 antibody	Potentiates innate immune responses in NSCLC patients.	[180]
HIF-1α	HIF-1α positively regulates PD-L1 expression. Targeting HIF-1α blocks hypoxic adaptation.	Anti-PD-L1 antibody	Improves prognosis and provides a precision therapy strategy.	[147]

**Note: Abbs.:** KEAP1, Kelch-like ECH-associated protein 1; PD-L1, Programmed Death-Ligand 1; DRP-104, Glutamine antagonist; PD-1, Programmed Cell Death Protein-1; CPT1A, Carnitine palmitoyltransferase 1A; FAO, Fatty Acid Oxidation; HDAC, Histone Deacetylase; MDSCs, Myeloid-derived suppressor cells; Arg-1, Arginase 1; iNOS, Inducible Nitric Oxide Synthase; YAP/TAZ, Yes-Associated Protein/Transcriptional Coactivator with PDZ-Binding Motif; TAMs, Tumor-Associated Macrophages; LUAD, Lung Adenocarcinoma; AMPK, AMP-Activated Protein Kinase; NSCLC, Non-Small Cell Lung Cancer; HIF-1α, Hypoxia-Inducible Factor 1 Alpha.

## 5 Discussion

Lung cancer progression is driven by a dynamic, bidirectional crosstalk between tumor cell metabolic reprogramming and immune suppression within the TME. This review has synthesized current evidence illustrating how metabolic adaptations in cancer cells (e.g., upregulated glycolysis, glutamine dependency, enhanced lipogenesis) not only fuel proliferation but also actively remodel the TME into an immunosuppressive niche. Conversely, immune cells within the TME undergo their own metabolic reprogramming, which often further supports tumor immune evasion. This reciprocal relationship establishes a positive feedback loop that promotes malignancy, therapeutic resistance, and poor patient outcomes. Despite substantial advancements in preclinical studies, numerous metabolic pathway-targeted therapies have yielded disappointing outcomes in LC clinical trials. This emphasizes the critical need for a more nuanced, systems-level comprehension of metabolism-immunity interactions.

### 5.1 Why Previous Metabolic-Targeted Therapies Mostly Failed in Clinical Trials

Early metabolic-targeted trials yielded disappointing results due to three critical oversights: (1) Tumor Heterogeneity: Metabolic heterogeneity presents a major therapeutic challenge in lung cancer [181]. Fundamental differences in metabolic profiles exist not only between subtypes [182] (LUAD/LUSC/SCLC) but also among tumors with similar clinical presentations, stemming from varied molecular drivers like genetic mutations and pathway activations [183]. This diversity directly undermines the effectiveness of metabolic-targeted approaches, resulting in disappointing clinical response rates. (2) Metabolic Adaptability: including metabolic flexibility and plasticity [184]. Metabolic flexibility denotes the ability of cancer cells to switch between different metabolic substrates in the nutrient-scarce TME. In contrast, metabolic plasticity refers to their capacity to bypass impairments in specific pathways, often by triggering compensatory mechanisms upon inhibition [185,186]. This adaptive response allows tumors to evade metabolic crises induced by targeted agents, ultimately limiting therapeutic benefit. (3) Clinical Strategy Gaps: Trials lacked patient selection based on metabolic vulnerability, faced on-target toxicities, and relied on inadequate biomarkers instead of direct TME validation. Future trials require spatial metabolomics and real-time metabolic imaging for patient stratification and target validation [187,188].

### 5.2 Controversies and Unresolved Questions in Metabolic-Immune Crosstalk

Persistent fundamental contradictions hinder understanding of lung cancer metabolism. The regulatory paradox of OXPHOS in cancer stems from the dichotomy of its dual “survival-function” roles. As an efficient energy supplier, it serves as both a key support for maintaining cancer stem cell (CSC) stemness and drug resistance, and a suppression target under microenvironmental stress (e.g., hypoxia). Meanwhile, it promotes tumor progression via ROS signaling yet may induce oxidative damage [189]. Similarly, glutamine supports tumor cell survival under hypoxic and glucose-deprived conditions by activating HIF-α and Akt/mTOR signaling pathways. In NSCLC, resistant cells rely on the glutamine metabolic enzyme GLUD1 to produce α-ketoglutarate (α-KG), which activates Snail expression to promote migration; targeting glutamine metabolism enhances sensitivity to EGFR-TKIs. Although glutamine predominantly promotes tumor progression, its deprivation or supplementation can suppress tumors in specific contexts. For example, dietary glutamine supplementation increases α-KG levels in the TME, reduces H3K4me3 methylation, and suppresses oncogenic pathways, while also reducing Treg differentiation and enhancing CD8^+^ T cell-mediated antitumor responses [190,191]. These paradoxes, shaped by tumor heterogeneity, metabolic plasticity, and dynamic microenvironmental changes, highlight that metabolic interventions are neither universally immunosuppressive nor stimulatory—their net effect depends on cell-specific metabolic wiring.

### 5.3 Clinical Translation: Overcoming Barriers to Precision Implementation

Successful translation from preclinical discovery to clinical application requires addressing three fundamental challenges. First, patient stratification must evolve beyond PD-L1 IHC to incorporate multi-omic signatures defining metabolic-immune TME subtypes, such as KEAP1-mutant NSCLC—a cohort with distinct glutamine dependency ideally suited for DRP-104 and anti-PD-1 combination therapy. Second, predictive biomarkers should extend beyond static genomics to include dynamic metabolic flux and spatial metabolomics [192–194]. Third, toxicity management for combinations demands innovative approaches like sequential scheduling (metabolic priming before ICI) and tumor-selective nanocarriers targeting specific receptors to minimize systemic toxicity while maximizing efficacy [195,196].

### 5.4 Refining the Framework for Metabolic-Immune Combination Therapies

We propose a stratified framework to stratify combination strategies by developmental maturity, addressing the current lack of stratification.

Clinically validated in lung cancer: Several combination therapies have reached clinical-stage testing in LC. Although grounded in a rational strategy of glutamine metabolic co-dependency, the combination of Telaglenastat (CB-839, 800 mg twice daily) and nivolumab exhibited a manageable safety profile in NSCLC patients; however, it showed no significant antitumor efficacy, potentially due to tumor heterogeneity or prior therapy resistance [197]. These findings inform future combination strategies targeting glutamine metabolism while necessitating further research to validate biomarker roles. The efficacy of such regimens, moreover, offers critical human data to validate the feasibility of metabolic immunotherapy.

Early-Phase Clinical: Strategies such as HDAC inhibitors (e.g., entinostat) combined with anti-PD-1 therapy are undergoing early-phase trials in multiple cancers [198,199]. Their potential lies in reversing epigenetic silencing of immune-related genes in T cells and myeloid cells. Yet, their efficacy in lung cancer hinges on biomarker identification—potentially derived from the metabolic subtypes outlined—to select responsive patients.

Preclinical: Numerous attractive metabolic nodes, such as PHGDH (serine pathway) [200] and CPT1A (fatty acid oxidation) [201], remain in the preclinical stage. While genetic knockdown studies confirm efficacy, the absence of specific, potent, and non-toxic small-molecule inhibitors for clinical application represents a significant barrier. Their role may be context-dependent, for instance, in tumors exhibiting defined lipid-metabolism signatures.

### 5.5 Precision Targeting through AI and Multi-Omics Integration

The integration of artificial intelligence (AI) with single-cell and spatial multi-omics is pivotal for decoding intratumoral metabolic-immune complexity, as AI/machine learning (ML) algorithms synthesize multi-omic data—genomic, transcriptomic, proteomic, and metabolomic—from single-cell RNA sequencing and spatial metabolomics to develop predictive models that optimize immunotherapy (e.g., ICIs + metabolically targeted agents) and guide combination therapies. Spatial transcriptomics reveals stark metabolic heterogeneity, where the hypoxic, glycolysis-dominated tumor core upregulates immunosuppressive markers (e.g., PD-L1), driving exhaustion of CTLs and NK cells and sparse immune infiltration, while the vascular-rich periphery exhibits greater infiltration of immune cells (e.g., CD8^+^ T cells, dendritic cells), forming an active interface often compromised by immunosuppressive cells like Tregs and MDSCs. This immune disparity, fueled by metabolic reprogramming, is addressed through metabolic-targeted therapies that modulate nutrient competition and reverse immunosuppression, with future efforts focused on integrating spatial multi-omics to devise personalized combinations—such as pairing immunotherapy with inhibitors of oxidative phosphorylation (OXPHOS) or lactate pathways—to improve prognostic outcomes [202,203].

## 6 Future Directions

Future research should now pivot from mechanism recapitulation to resolving key dilemmas: the optimal sequencing of metabolic modulators with immunotherapy, managing on-target toxicities against essential immune metabolic pathways, and expanding beyond Warburg effect to explore micronutrients and the microbiome. In conclusion, despite the heightened complexity revealed, the pursuit of targeting cancer metabolism remains sound. The failure of early monotherapies highlights that success will depend on a nuanced grasp of TME heterogeneity, the development of precise biomarkers, and the rational design of combination therapies, empowered by technological advances like AI and single-cell multi-omics to achieve clinical translation.

## 7 Conclusion

Lung cancer progression is governed by a dynamic metabolic-immune axis, defying simplistic intervention. Future advances in targeting the lung cancer metabolic landscape require a definitive shift from non-selective strategies to biomarker-guided precision. Success hinges on leveraging AI and multi-omics to decipher heterogeneity and stratify patients based on metabolic-immune phenotypes, ultimately unlocking the potential of metabolic-immunotherapy.

## Data Availability

Not applicable.
